# Physical Activity and Exercise for Cardiorespiratory Health and Fitness in Chronic Kidney Disease

**DOI:** 10.31083/j.rcm2308273

**Published:** 2022-07-26

**Authors:** Jared M. Gollie, Scott D. Cohen, Samir S. Patel

**Affiliations:** 1 Research & Development Service, VA Medical Center, Washington DC 20422, USA; 2 Department of Health, Human Fuction, and Rehabilitation Sciences, The George Washington University, Washington DC 20037, USA; 3 Renal Service, VA Medical Center, Washington DC 20422, USA; 4 Department of Medicine, The George Washington University, Washington DC 20037, USA

**Keywords:** aerobic exercise, resistance exercise, cardiorespiratory fitness, strength, vascular function, oxygen consumption

## Abstract

Chronic kidney disease (CKD) is associated with an increased risk for cardiovascular disease (CVD), major adverse CVD events, and cardiovascular mortality. Low levels of physical activity and reduced cardiorespiratory fitness further compound the health consequences in this patient population. Aerobic exercise alone and the combination of aerobic and resistance exercise have beneficial effects for improving aerobic capacity while resistance exercise alone improves strength and skeletal muscle health. Given the prevalence of CVD in CKD patients and limited treatment options targeting traditional and non-traditional CVD risk factors in this population, the incoroporation of physical activity and exercise into the care of CKD seems critical for improving patient outcomes. Therefore, the purpose of this narrative review is to discuss the evidence of physical activity and exercise in CKD patients and the effects on cardiovascular outcomes and fitness.

## 1. Introduction

An estimated 10–15% of the global population is living with chronic kidney disease (CKD) [1]. The majority of adults with CKD are unaware of their condition including two in five adults with severe CKD [2]. The classification of CKD is based on overall kidney function and determined by measures of estimated glomerular filtration rate (eGFR) and or markers of kidney damage [1,3]. Stage G1 reflects grossly intact kidney filtration, or normal eGFR, but with a marker(s) of kidney damage (e.g., proteinuria), whereas stage G5 reflects almost complete loss of kidney filtration (i.e., kidney failure) with an eGFR of less than 15 mL/min/1.73 m^2^ (Table 1, Ref. [3]). Type II diabetes mellitus and hypertension are two of the most common causes of CKD, with the incidence of CKD being greatest in adults 65 years of age and older [2]. Metabolic derangements including anemia, hyperparathyroidism, metabolic acidosis, sodium retention, and hyperkalemia increase in prevalence and severity with CKD progression below an eGFR of 60 mL/min per 1.73 m^2^. Many of these derangements are clinically apparent by an eGFR 45 mL/min per 1.73 m^2^ or less; and most, if not all, are present and require treatment by eGFR <30 mL/min per 1.73 m^2^.

The risk for cardiovascular events are significantly higher in patients with CKD [4]. Patients with CKD G3 not on dialysis are at greater risk of mortality caused by cardiovascular disease (CVD) than mortality caused by kidney disease [4]. Approximately 50% of all patients with CKD G4 and G5 have CVD, which is also the number one cause of death in this group of patients [1,5,6]. CKD patients experience a higher risk of myocardial infarction, stroke, congestive heart failure and arrhythmias [7]. The rates of traditional CVD risk factors are elevated in the CKD patient population including diabetes mellitus, hypertension, and hyperlipidemia [8]. However, this accounts for only part of the greater risk for CVD in the CKD patient population. Moreover, randomized controlled trials examining the effects of interventions on traditional and non-traditional CVD risk factors in CKD patients have demonstrated little benefit to date [9–12].

Reductions in kidney function result in decreased excretion and degradation of advanced glycation end products and small inflammatory molecules creating a pro-inflammatory state [13–16]. Additional factors including uremia, diet, lifestyle, metabolic acidosis, vitamin D deficiency as well as the production of cytokines from other organs, also contribute to elevations in systemic inflammation observed in CKD [17]. Circulating concentrations of biomarkers of inflammation are found to be inversely associated with measures of kidney function and directly associated with albuminuria [18,19]. Chronic inflammation in CKD contributes to vascular and myocardial remodeling processes resulting in atherosclerotic lesions, vascular calcification, and vascular senescence in addition to myocardial fibrosis and calcification of cardiac valves [20,21]. Moreover, the uremic milieu can lead to phenotypic changes in vascular smooth muscle cells causing them to switch to osteoblast-like cells [22]. The resulting medial vascular calcification affects central arterial blood vessels leading to increases in cardiac afterload and pulse wave velocity (PWV), and worsening congestive heart failure. The calcification can also affect cardiac valves, particularly the aortic valve. CKD is also associated with unique changes in the myocardial wall including increased myocardial fibrosis and cardiac hypertrophy [23]. Left ventricular hypertrophy is present in approximately one-third of CKD patients and three-quarters of patients with end-stage kidney disease (ESKD) [23].

Physical activity and exercise are regarded as two of the most effective ways to improve health and function [24–26]. In a metaepidemiological study comparing meta-analyses of randomized controlled trials, exercise interventions were found to have similar, if not superior, benefits on mortality, secondary prevention of coronary heart disease, treatment of heart failure, and prevention of diabetes when compared to medications [27]. Reductions in medication use has also been shown over the course of twelve-months of supervised exercise [28]. In a landmark study, Myers *et al*. [29] reported that peak exercise capacity was the strongest predictor of risk of death among adults with and without CVD. Given the prevalence of CVD in CKD patients and limited treatment options targeting traditional and non-traditional CVD risk factors in this population, the incoroporation of physical activity and exercise into the care of CKD seems critical for improving patient outcomes [30,31]. Therefore, the purpose of this narrative review is to discuss the evidence of physical activity and exercise in CKD patients and the effects on cardiovascular outcomes and fitness.

## 2. Physical Activity

Physical activity describes any bodily movement produced by skeletal muscles that results in an increase in caloric requirements over resting energy expenditure [25]. Conversely, exercise refers to a type of physical activity consisting of planned, structured, and repetitive bodily movement to improve and or maintain one or more components of physical fitness [25]. General physical activity guidelines for adults with chronic health conditions include engaging in at least 150 minutes (2 hours and 30 minutes) to 300 minutes (5 hours) a week of moderate-intensity, or 75 minutes (1 hour and 15 minutes) to 150 minutes (2 hours and 30 minutes) a week of vigorous-intensity aerobic physical activity, or an equivalent combination of moderateand vigorous-intensity aerobic physical activity [25,32]. In addition, adults are encouraged to also perform musclestrengthening activities of moderate or greater intensity involving all major muscle groups on 2 or more days per week [25,32]. If an individual is unable to meet the recommended guidelines of physical activity described above, they are encouraged to increase time spent performing physical activity in accordance with their abilities while avoiding physical inactivity [25,32].

Lack of physical activity is a primary contributor to chronic disease [33–35]. Evidence strongly supports that high amounts of sedentary behavior increase the risk for all-cause and CVD mortality and type II diabetes [36–39]. For example, an inverse, non-linear dose-response relationship has been oberserved between long-term leisure-time physical activity and all-cause and CVD mortality when assessed up to 23-years of follow-up [39]. Exercise capacity and energy expenditure were found to be stronger predictors of mortality than smoking, hypertension, obesity, and diabetes [40]. According to the Centers for Disease Control and Prevention (CDC), only one in four adults meet the recommended levels of physical activity [32]. The annual health care costs of physical inactivity in the United States alone is estimated to be $117 billion [2]. Physical inactivity related deaths contributed to $13.7 billion in productivity losses, and physical inactivity was responsible for $13.4 billion disability-adjusted life-years [41].

High levels of physical inactivity are reported in the CKD population, with physical inactivity levels increasing with disease progression [42–46]. Using the National Health and Nutrition Examination Survey III (NHANES III), 28% of individuals with CKD were reported to be physically inactive as compared to 13.5% of non-CKD individuals [47]. Consistent with findings in non-CKD adults, low levels of physical activity are associated with all-cause mortality and CVD events in individuals with CKD [47–51]. In maintenance hemodialysis patients, engaging in habitual physical activity was associated with decreased risk for mortality over a median follow-up of 45-months [52]. Physical activity level is also shown to be associated with cardiovascular mortality and all-cause mortality in kidney transplant recipients [49–51]. Using a modified Yale Physical Activity Survey, Kang *et al*. [49] reported that kidney transplant recipients in the highest tertile of physical activity experienced significantly lower risk of CVD events, CVD mortality, and all-cause mortality. Similarly, greater pretransplant physical activity is found to be strongly associated with better survival during a median follow-up period of 8-years in kidney transplant recipients [51].

Correlates of physical inactivity in adults with CKD include being older, female, smoking, having a greater number of comorbidities, and low level of education [42, 43,45,51]. In kidney transplant recipients, physical activity is inversely associated with metabolic syndrome, history of CVD, fasting insulin, and triglyceride concentration; and positively associated with kidney function and 24-hour urinary creatinine excretion when adjusting for age [50]. Across all CKD stages, higher serum albumin, creatinine, cardiorespiratory fitness, and self-efficacy levels, and lower body mass index are found to be associated with greater physical activity [42]. In dialysis patients, number of steps taken is positively associated with body compositional measures (i.e., body water, fat mass, body mass index, lean body mass, intracellular water, phase angle), serum albumin, hematocrit, and hemoglobin [44]. Differences are also reported between type of dialysis treatment, with patients in hemodialysis using a catheter reporting lower levels of physical activity compared to patients on peritoneal dialysis, hemodialysis using an arteriovenous fistula, or with a graft [43]. The relationship between physical activity level and hemoglobin concentration is less clear in CKD patients, with one study reporting no association [43] while other studies found that higher hemoglobin concentration was associated with increased levels of physical activity [42,44].

Physical activity levels directly affect physical function [53,54] and are hypothesized to impact metabolic adaptations to exercise [55]. Adults reporting 150 minutes or more of moderate physical activity per week were found to have an average physical function score, as determine by the physical function subscale from the 36-item Short Form Questionnaire, *≈*20 points higher than those not meeting this recommendation [54]. Lower activity scores are associated with poor physical function and mental health in dialysis patients [43,48]. Importantly, higher physical activity levels are found to be associated with slower decline in eGFR in patients with established CKD and older adults [56,57]. In 4011 ambulatory participants aged 65 or older without CKD, the estimated risk of rapid decline in kidney function was greatest in individuals with lower physical activity levels [56]. In adults with CKD not requiring dialysis, greater than 150 minutes of physical activity per week had the lowest rate of loss in cystatin C based-eGFR [57]. Moreover, each 60-minute increment in weekly physical activity duration was associated with a 0.5% slower decline per year in eGFR during a median follow-up of 3.7 years [57]. In patients who experienced a recent acute myocardial infarction, renal function evaluated using cystatin C based-eGFR significantly increased in patients with high physical activity levels (7102 ± 2365 steps/day) when compared to those with low physical activity levels (2335 ± 1219 steps/day) over the course of 3 months [58].

Using the American College of Sports Medicine guidelines, patients with CKD G3 and G4 significantly improved metabolic equivalents (METs), 6-minute walk distance, and body mass index following 12-months of exercise [59]. Physical activity levels significantly increased at 6-months but decreased to baseline at 12-months [59]. In G3 and G4 CKD patients, a 3-year multidisciplinary lifestyle intervention increased the number of patients meeting the physical activity guideline target of 500 METs minutes per week from 29% to 63%. At 12-months both peak oxygen consumption (VO_2peak_) and METs increased significantly by 9.7% and 30%, respectively [60]. Of note, METs remained elevated at 24- and 36-months despite VO_2peak_ returning to near baseline levels [60]. Other studies, however, reported no change in physical activity status of CKD patients in response to exercise [61–63]. Therefore, future studies are warranted to determine the effects of exercise for improving physical activity levels in CKD patients and the potential interactions between physical activity level and adaptations to exercise.

## 3. Aerobic Exercise

Cardiorepiratory fitness is a strong predictor of future major adverse cardiovascular events and CKD incidence [64–66]. Cardiorespiratory fitness is often severely compromised in CKD patients [67]. Howden *et al*. [67] noted a 17% reduction in VO_2peak_ in patients with mild-to-moderate CKD when compared to age-predicted values. In CKD patients not on dialysis, aerobic capacity has been found to be significantly associated with eGFR [68]. The incidence of CKD over a median follow-up of 7.9 years was observed to be inversely related to exercise capacity [66]. In a multivariable-adjusted proportional hazards model, individuals classified as moderately or highly fit had a 24% and 34% lower risk of developing CKD when compared to those classified as low fit [65]. In ambulatory ESKD patients, VO_2peak_ is a strong predictor of survival when examined over a period of approvimately 3.5 years [69]. Similarly, in kidney transplant recipients, mortality risk in patients with at least 3-years post-transplant follow-up is greatest in those with low VO_2peak_ [70].

Cardiorespiratory fitness is determined by the heart, blood vessels, lungs, and skeletal muscles ability to transport and utilize oxygen during physical activity [71–73]. In young and healthy populations, maximal oxygen consumption (VO_2max_) is predominantly limited by central circulatory processes responsible for oxygen delivery whereas peripheral processes involved in oxygen utilization determine cardiorespiratory endurance [72,74]. However, in older adults and chronic disease states in which oxygen delivery is reduced or impaired, oxygen delivery plays a greater role in cardiorespiratory endurance [75–77]. In CKD patients, lower cardiorespiratory fitness likely results from limitations in oxygen delivery and utilization [73,78–81]. In non-dialysis CKD patients, exercise capacity was strongly associated with oxygen delivery, and specifically peak heart rate [82]. Moore *et al*. [83] reported that ESKD patients achieved 77% of predicted peak heart rate during exercise testing with reduced hemotrocrit measured at rest which also suggests the presence of an oxygen delivery limitation. Others found PWV to be one of the strongest independent determinants of VO_2peak_ in CKD patients [68]. In support of potential peripheral limitations, low muscle oxygen conductance has been reported in CKD patients [84,85].

Aerobic exercise recommendations for people with CKD are presented in Table 2 [25,30]. Available data suggests aerobic exercise improves VO_2peak_ in patients with CKD, ESKD, and kidney transplant recipients [61,79,86–95]. The magnitude of aerobic exercise-induced improvement in VO_2peak_ is estimated to be *≈*2 mL·kg^−1^·min^−1^ [79,87,88,96]. Aerobic exercise has also been shown to elicit positive effects on exercise duration (i.e., endurance), health-related quality of life (HRQoL), and physical function [87,88,93,97–100]. Despite the noted increases in VO_2peak_ in response to exercise, aerobic capacity still remins below normative values of sedentary non-CKD adults [78,79]. Moreover, the effects of aerobic exercise on cardiovascular outcomes is less clear in the CKD population [63,95,97,101,102]. For example, no changes were observed in measures of vascular function following 3-months of moderate-intensity aerobic exercise in G3 and G4 CKD patients when assessed via flow mediated dialation of the brachial artery, carotid-femoral PWV, or cellular markers [97]. In a meta-analysis, only marginal differences were observed in heart rate maximum with no statistical differences found in exercise capacity, blood pressure, resting heart rate, serum lipid, and serum creatinine between aerobic exercise and controls [87]. The lack of differences were still present regardless of outcome when studies were divided based on exercise intensity, treatment of dialysis, or length of intervention [87]. Conversely, Kirkman *et al*. [103] demonstrated improved microvascular function while maintaining conduit artery function after 12-weeks of aerobic exercise in CKD patients not on dialysis. Regarding peripheral adaptations to aerobic exercise, ESKD patients who demonstrated improvements in VO_2peak_ following 12-weeks of training also experienced widening of their arterio-venous oxygen difference in the absence of change in central adaptations [81].

Due to the prevalence of anemia in the CKD population [104], several studies have investigated the use of erythropoietin as a possible treatment for enhancing aerobic capacity [85,105,106]. Stray-Gundersen *et al*. [106] examined the effects of an erythroid-stimulating agent and aerobic exercise training on aerobic capacity in hemodialysis patients. Both hemotocrit normalization and aerobic exercise increased VO_2peak_, however, the improvement did not reach the level of normative values [106]. When the perturbations were analyzed independently, hemotocrit normalization increased peak arterial oxygen and arterio-venous oxygen difference, whereas exercise improved cardiac output, citrate synthase activity, and peak diffusing capacity [106]. Reductions in muscle blood flow have been shown to accompany the increases in hemoglobin concentration with erythropoietin in CKD, suggesting low muscle oxygen conductance [84,85]. Evidence from muscle biopsies of the vastus lateralis showed thickening of the endothelium and electron-dense interstitial deposits further supporting that the inability to normalize exercise capacity may be caused by abnormalities within skeletal muscle [106].

## 4. Resistance Exercise

Reductions in kidney function place patients with CKD at an increased risk for neuromuscular impairments [107–109]. The rapid loss in skeletal muscle in the presence of CKD is suggested to occur as a result of several processes upregulating protein degradation while simultaneously downregulating protein synthesis [107]. Low muscle mass and strength are associated with the development of CVD, CVD mortality, and CVD-related outcomes [110]. In patients with CKD, those with low psoas muscle mass had significantly higher risk of major adverse cardiovascular events compared to patients with high psoas muscle mass during a median follow-up period of 3.2 years [111]. Moreover, low psoas muscle mass was found to be an independent predictor of major adverse cardiovascular events in these CKD patients [111].

Resistance exercise may also assist in protecting against CVD and CVD risk factors. For example, men with high levels of muscular strength during adolescences have a decreased risk for CVD disease later in life while low muscular strength is associated with increased risk of mortality during middle age [112]. In addition, muscular strength is shown to have an independent protective effect on all-cause and cancer mortality in healthy middle-aged men, as well as in men with hypertension and patients with heart failure [113]. Age-related weight and adiposity gains, risk of hypertension, and prevalence and incidence of metabolic syndrome were all found to be inversely associated with muscular strength [113]. Importantly, muscular fitness maintains its protective effects even when considering an individual’s cardiorespiratory fitness [113]. Higher levels of muscular fitness may, to some extent, counteract the adverse cardiovascular profile of overweight and obese individuals [113,114]. Therefore, the ability of resistance exercise to increase lean mass, decrease fat mass, and improve glycemic control and blood lipid profiles would be advantageous for reducing CVD risk in patients with CKD [115–117].

Resistance exercise describes any activity capable of overloading the neuromuscular system in an attempt to preserve or improve neuromuscular health [118]. Resistance exercise is an effective approach for addressing functional deficits experienced with aging and disease [119, 120]. Weekly resistance exercise performed one, two, or three times or total amount of 1–59 minutes, is associated with approximately a 40–70% decreased risk of total CVD events, independent of aerobic exercise [114]. Similar results are observed for CVD morbidity and all-cause mortality [114]. Resistance exercise is found to indirectly lowered CVD risk by decreasing body mass index [114]. Resistance exercise, even less than 1 hour per week, is associated with a lower risk of development of metabolic syndrome over a median follow-up of 4 years, independent of aerobic exercise [121].

Resistance exercise recommendations for people with CKD are provided in Table 3 [25,30]. Given the increase susceptibility for neuromuscular impairments with CKD, resistance exercise has been proposed as a potential treatment option for maintaining neuromuscular health [108]. To determine the impact of resistance exercise in patients with moderate CKD, Castaneda *et al*. [122] conducted an elegant study in which resistance exercise plus low-protein diet was compared to low-protein diet alone for 12-weeks. The patients who were randomized to the resistance exercise plus low protein group increased total body potassium, type I and II muscle fiber cross-sectional area, and muscle strength while maintaining their body weight whereas patients on the low-protein diet only did not [122]. Therefore, even in the presence of low-proetin intake, resistance exercise seems to counteract catabolism in CKD patients [122]. Evidence to date suggests that progressive resistance exercise is an effective approach for inducing muscle hypertrophy and improving muscle force capacity, aspects of physical functioning, and health-related quality of life in patients with CKD [89,123,124]. Similar findings are reported when implementing resistance exercise during dialysis treatment [125–127]. However, resistance exercise does not seem to have a direct affect on aerobic capacity in CKD patients [89].

## 5. Combination of Aerobic Exercise with Resistance Exercise

Combination exercise describes combining both aerobic exercise and resistance exercise performed within the same exercise session or on alternating days at dosages meeting the recommended physical activity guidelines [25, 26]. While both aerobic exercise and resistance exercise performed independently demonstrate positive health and functional outcomes in CKD patients, the greatest benefits from exercise may be achieved when these exercise modes are incorporated together. Combination exercise is reported to increase aerobic capacity in CKD patients, with the potential for greater effects than aerobic exercise alone [89]. Several studies have documented a slowing in the decline of kidney function, and in some instances improvements in kindey function, in response to combination exercise [128–130]. For example, Nylen *et al*. [129] reported that patients with CKD G2 and G3 significantly improved eGFR following 12-weeks of supervised aerobic and resistance exercise. While combination exercise shows promise for the potential to slow the decline in eGFR and possibly improve eGFR, additional large randomized well-controlled studies are required to truly determine the impact of combination exercise on kidney function.

In CKD patients on dialysis, signficant improvements in VO_2peak_, depressive symptoms, and health-related quality of life are reported when combination exercise is implemented during dialysis treatment sessions. Combination exercise (30–60 min cycling, 20 min strengthening) performed 3 times per week during the first 2 hrs of hemodialysis over 12-months improved VO_2peak_ from 16.8 mL·kg^−1^·min^−1^ to 22.3 mL·kg^−1^·min^−1^ [131]. Similarly, when comparing combination exercise to aerobic exercise alone performed during dialysis treatment sessions, VO_2peak_ increased to a greater extent in the combination exercise group [132]. In non-diabetic adult patients with hypertension and CKD G2–G4, 16-weeks of combination exercise significantly decreased high-sensativity C-reactive protein (hs-CRP) and fasting blood glucose while improving functional capacity (i.e., senior fitness test, 30-second sit-to-stand, 2-min step test, 8-foot up and go) [133].

When compared to aerobic exercise alone, 12-weeks of combination exercise in CKD patients not on dialysis (G3b–G5) significantly increased knee extensor strength and quadriceps muscle volume [134]. Interestingly, neither aerobic exercise alone or combination exercise was found to improve VO_2peak_ in these CKD patients [134]. In a follow-up study, the effects of aerobic exercise alone and combination exercise on mitochondrial outcomes were examined to better understand the lack of improvements seen in VO_2peak_ [135]. Neither aerobic exercise alone nor the combination of aerobic exercise with resistance exercise was able to reverse deficits in mitochondrial mass or gene expression of transcription factors involved in mitochondrial biogenesis in CKD patients [135]. In a recent large randomized controlled trial, 6-months of intradialytic cycling performed 3 times per week combined with resistance exercise twice per week failed to improve VO_2peak_ or functional outcomes [136]. These findings highlight the need for additional studies to determine which factors are most critical for eliciting meaningful exercise-induced adaptations across all stages of CKD.

## 6. Effects of Physical Activity and Exercise on Inflammation in CKD

Chronic low-grade systemic inflammation, characterized by high circulating levels of pro-inflammatory markers, is posited as both a strong risk factor for and pathogenic mechanism in CVD [137]. In addition to CVD, inflammatory status is implicated in declines in functional capacity and skeletal muscle wasting in CKD patients [107,138–140]. Greater amounts of physical activity are associated with lower inflammatory status [141]. Both hysical activity and exercise elicit beneficial effects on inflammation through the promotion of an anti-inflammatory state [142,143]. Reduction in visceral fat mass and induction of an anti-inflammatory environment with each exercise bout have been suggested to mediate the anti-inflammatory effects of regular exercise [142]. In CKD patients, a large inflammatory response was observed to an acute bout of unaccustomed resistance exercise evidenced by significantly elevated levels of interleukin-6 (IL-6), monocyte chemoattractant protein-1 (MCP-1), and tumor necrosis factor-alpha (TNF-*α*) [144]. Importantly, this inflammatory response was reduced following 8-weeks of progressive resistance exercise [144]. Similarly, 12-weeks of progressive resistance exercise combined with low-protein diet reduced serum CRP and IL-6 [145]. Reduction in the ratio of plasma IL-6 to interleukin-10 (IL-10) levels and downregulation of T-lymphocyte and monocyte activation have also been observed following 6-months of regular walking in CKD patients [146]. While these findings support the potential benefits of exercise on inflammatory status, other studies have reported no change in inflammatory markers in response to exercise in those with CKD [140,147].

## 7. Barriers and Facilitators to Physical Activity and Exercise in CKD Patients

To better understand the lack of physical activity and exercise in CKD patients, several studies have examined which factors act as barriers and facilitators to physical activity and exercise participantion. Using the COM-B model of the Behavior Change Wheel (BCW) in ESKD patients, Clarke *et al*. [148] identified poor physical condition and symptoms associated with the patient’s disease and treatment (i.e., reduced walking ability, fatigue, tiredness, pain, shortness of breath, lower extremity ulcers, and weakness), as well as existing musculoskeletal injuries or concerns, previous surgeries, and co-morbidities as barriers to the performance or promotion of exercise. Importantly, a lack of awareness of exercise guidelines was indicated as a barrier to exercise by both patients and health care providers (HCP) [148]. Other barriers identified include lack of accessibility, fatigue on dialysis days and non-dialysis days, shortness of breath, lack of motivation, endorsement of too many medical problems, and not having enough time on dialysis days [148–154]. Facilitators to physical activity performance or promotion, on the other hand, include encouragement from HCP’s, friends and family, peer support, feeling healthy, wanting to feel better, wanting to improve health, wanting to enhance physical mobility, and wanting to increase strength.

Given the barriers described above, excessive levels of fatigability when engaging in physical activity may pose a significant challenge to meeting the recommended physical activity and exercise guidelines in CKD patients [155]. While achieving the physical activity and exercise guidelines may not always be feasible in those with CKD, any increase in the amount of daily physical activity while reducing the sedentary time is likely to have profound health benefits [156]. It has been recommended that kidney care programs or dialysis centers offer patient physical activity or exercise plans prescribed by an exercise professional (physical therapist, kinesiologist, physiotherapist, exercise physiologist) to increase physical activity and exercise participation [148]. One exercise prescription strategy which may be advantageous is the sequencing of exercise modes targeting the development of neuromuscular force capacity prior to cardiorespiratory fitness. Such approaches have been suggested for frail and sarcopenic populations in which a period of resistance exercise is necessary for the development of neuromuscular capacity prior to engaging in aerobic exercise [157,158]. Even though resistance exercise has not been shown to have a direct impact on aerobic capacity in CKD patients, skeletal muscle mass and strength may have important implications for the amount and intensity of aerobic exercise prescribed. Considering that strength is significantly associated with VO_2peak_ in both ESKD patients and kidney transplant recipients [159,160], the sequencing of resistance exercise followed by aerobic exercise could provide a useful strategy for improving cardiorespiratory fitness. However, the efficacy of exercise regimens structured to develop neuromuscular capacity prior to targeting aerobic capacity still remains an unanswered question in the CKD population and therefore requires additional research.

## 8. Future Research Considerations

The associations between physical activity and CVD, CVD mortality and all-cause mortality in CKD patients underscores the importance of maintaining an active lifestyle. Furthermore, existing data supports the potential for exercise interventions to improve cardiorespiratory fitness and aspects of cardiovascular health. However, before we are fully able to appreciate the benefits of physical activity and exercise in the CKD population, several key areas require further investigation. Understanding the exact mechanisms underlying adaptations to exercise will help inform the design of future interventions. The development of physical activity and exercise guidelines specifically for CKD patients should be prioritized. Information regarding the interactions between physical activity status and adaptations to exercise interventions in CKD patients is currently lacking and may help explain differences in responses to exercise across patients. Optimal sequencing of exercise modes and exercise dosing to target specific health and functional outcomes in CKD patients has yet to be established. The effects of physical activity and exercise on cognitive function in CKD patients is currently unclear. Additionally, strategies for monitoring physical activity in clinical settings and increasing physical activity participation and adherence should be examined.

## 9. Summary

Physical inactivity presents major challenges to overall health and function in CKD patients. Engaging in structured exercise may assist CKD patients in meeting the recommended amounts of physical activity required for the maintenance or improvement of health. Even if CKD patients are unable to meet current physical activity guidelines, increasing activity levels while minimizing sedentary time could have meaningful health implications. Aerobic exercise alone and the combination of aerobic exercise and resistance exercise seem effective for increasing cardiorespiratory fitness as measured by VO_2peak_ although values do not reach normative levels. Resistance exercise is more effective for improving strength and neuromuscular characteristics. While physical activity and exercise are encouraged, the exact mechanisms underlying the adaptations to exercise in CKD patients are not entirely clear. Furthermore, the most effective approaches for eliciting specific health and functional outcomes in CKD patients have yet to be determined. Therefore, future research should focus on establishing CKD-specific physical activity and exercise recommendations in accordance with the underlying pathophysiology and unique exercise responses observed in this patient population.

## Figures and Tables

**Table 1.** Prognosis of chronic kidney disease (CKD) and albuminuria according to Kidney Disease: Improving Global Outcomes (KDIGO).

				Persistent albuminuria categories		
				Description and range		
				A1	A2	A3
				Normal to mildly increased	Moderately increased	Severely increased
				<30 mg/g	30–300 mg/g	>300 mg/g
				<3 mg/mmol	3–30 mg/mmol	>30 mg/mmol
eGFR categories (mL/min/1.73 m^2^)	G1	Normal	≥90			
	G2	Mildly decreased	60–89			
	G3a	Mildly to moderately decreased	45–59			
Description and range	G3b	Moderately to severely decreased	30–44			
	G4	Severely decreased	15–29			
	G5	Kidney failure	<15			

Abbreviations. eGFR, estimated glomerular filtration rate.

Green: low risk (if no other markers of kidney disease, no CKD); Yellow: moderately increased risk; Orange: high risk; Red: very high risk (used with permission from Kidney Disease: Improving Global Outcomes (KDIGO) CKD Work Group. KDIGO 2012 Clinical Practice Guideline for the Evaluation and Management of Chronic Kidney Disease. *Kidney inter., Suppl*. 2013; **3**: 1–150 [3]).

**Table 2.** Aerobic exercise recommendations for adults with kidney disease [25,30].

	Aerobic exercise
Frequency	3–5 d·wk^−1^
Intensity	Moderate intensity (40%−59% VO_2_R, RPE 12–13 on a scale of 6–20).
Time	20–60 min of continuous activity; however, if this cannot be tolerated, use 3–5 min bouts of intermittent exercise aiming to accumulate 20–60 min·d^−1^.
Type	Prolonged, rhythmic activities using large muscle groups (e.g., walking, cycling, swimming).

RPE, rating of perceived exertion; VO_2_R, oxygen consumption reserve.

**Table 3.** Resistance exercise recommendations for adults with kidney disease [25,30].

	Resistance exercise
Frequency	2–3 d·wk^−1^
Intensity	65%−75% 1-RM. Performance of 1-RM is not recommended unless medically cleared for such effort; instead, estimate of 1-RM from a ≥3-RM test.
Time	A minimum of 1 set of 10–15 repetitions, with a goal in most individuals to achieve multiple sets. Choose 8–10 different exercises targeting the major muscle groups.
Type	Machines, free weights, or bands.

1-RM, one repetition maximum; 3-RM, three repetition maximum.
